# Dual-Specificity Phosphatase 10 Controls Brown Adipocyte Differentiation by Modulating the Phosphorylation of P38 Mitogen-Activated Protein Kinase

**DOI:** 10.1371/journal.pone.0072340

**Published:** 2013-08-20

**Authors:** Hye-Ryung Choi, Won Kon Kim, Eun Young Kim, Baek Soo Han, Jeong-Ki Min, Seung-Wook Chi, Sung Goo Park, Kwang-Hee Bae, Sang Chul Lee

**Affiliations:** 1 Research Center for Integrated Cellulomics, KRIBB, Daejeon, Republic of Korea; 2 Department of Functional Genomics, University of Science and Technology (UST), Daejeon, Republic of Korea; 3 Biomedical Proteomics Research Center, KRIBB, Daejeon, Republic of Korea; Tohoku University, Japan

## Abstract

**Background:**

Brown adipocytes play an important role in regulating the balance of energy, and as such, there is a strong correlation between obesity and the amount of brown adipose tissue. Although the molecular mechanism underlying white adipocyte differentiation has been well characterized, brown adipocyte differentiation has not been studied extensively. Here, we investigate the potential role of dual-specificity phosphatase 10 (DUSP10) in brown adipocyte differentiation using primary brown preadipocytes.

**Methods and Results:**

The expression of DUSP10 increased continuously after the brown adipocyte differentiation of mouse primary brown preadipocytes, whereas the phosphorylation of p38 was significantly upregulated at an early stage of differentiation followed by steep downregulation. The overexpression of DUSP10 induced a decrease in the level of p38 phosphorylation, resulting in lower lipid accumulation than that in cells overexpressing the inactive mutant DUSP10. The expression levels of several brown adipocyte markers such as PGC-1α, UCP1, and PRDM16 were also significantly reduced upon the ectopic expression of DUSP10. Furthermore, decreased mitochondrial DNA content was detected in cells expressing DUSP10. The results obtained upon treatment with the p38 inhibitor, SB203580, clearly indicated that the phosphorylation of p38 at an early stage is important in brown adipocyte differentiation. The effect of the p38 inhibitor was partially recovered by DUSP10 knockdown using RNAi.

**Conclusions:**

These results suggest that p38 phosphorylation is controlled by DUSP10 expression. Furthermore, p38 phosphorylation at an early stage is critical in brown adipocyte differentiation. Thus, the regulation of DUSP10 activity affects the efficiency of brown adipogenesis. Consequently, DUSP10 can be used as a novel target protein for the regulation of obesity.

## Introduction

Adipose tissue is loose connective tissue that is primarily composed of adipocytes. It plays a critical role in energy homeostasis as both an energy reserve and an endocrine organ in animals [1]; [2]. Obesity is becoming a common health problem across the world. It occurs due to an imbalance between energy intake and energy expenditure and depends on the amount of body fat, specifically adipose tissue. Adipogenesis, the process by which a cell differentiates into an adipocyte, is highly regulated by a variety of mechanisms, including endocrine factors. In mammals, there are two types of adipocytes, white and brown adipocytes, which have opposite functions with regard to their energy balance. White adipocytes store excess energy as triglycerides in lipid droplets, whereas brown adipocytes release energy in the form of heat through thermogenesis. Brown adipocytes are very prominent in rodents and human infants [3]. However, brown adipocytes are increasingly thought to be important in adult humans. The amount of brown adipose tissue (BAT) in adult humans has been found to be highly correlated with their degree of obesity, indicating that brown adipocytes may play an important role in the prevention and treatment of obesity [3]–[6]. Therefore, a deeper understanding of the cellular and molecular mechanisms underlying brown adipocyte differentiation is required to treat and overcome obesity. Compared to the mechanisms related to white adipocytes, the molecular mechanisms underlying brown adipocyte differentiation have not been extensively investigated because this process was identified only recently in adult humans.

The p38 mitogen-activated protein kinases (MAPKs) are responsive to many stress stimuli such as cytokines, UV irradiation, and heat shock. They are involved in the processes of cell differentiation, apoptosis, and autophagy [7]–[9]. Dual-specificity MAPK kinase 3 (MKK3) and SAPK/ERK kinase (SEK) activate p38 MAPK by phosphorylation at Thr-180 and Tyr-182 [10]. Several studies have shown the involvement of p38 MAPK in adipocyte differentiation, although their results regarding the functional role of p38 have been somewhat contradictory. For example, Engelman *et al.* reported the positive role of p38 in adipogenesis in experiments using a p38 inhibitor [11], whereas another study showed that the addition of a p38 inhibitor at an early stage of adipogenesis induces decreased adipocyte differentiation by downregulating the phosphorylation of the transcription factor C/EBPβ and its post-translational activation [12]. Furthermore, Hata *et al*. showed that bone morphogenic protein-2 (BMP-2) induced pluripotent mesenchymal cell lines C3H10T1/2 into the adipocyte lineage through p38 activation [13]. For brown adipocyte differentiation, BMP-7 increases this differentiation by enhancing mitochondrial biogenesis via p38 [14]. In addition, cardiac natriuretic peptides act via p38 to induce the brown fat thermogenic program [15]. These reports clearly imply that p38 is involved in brown adipocyte differentiation.

Protein phosphorylation is a common post-translational modification that regulates a wide range of the signaling pathways involved in differentiation, apoptosis, proliferation, gene regulation, and metabolism. Although phosphorylation at tyrosine occurs less frequently than phosphorylation at serine and threonine, tyrosine phosphorylation is very important because it occurs almost exclusively in multicellular eukaryotes [16]–[18]. The balanced action between protein tyrosine kinases (PTKs) and protein tyrosine phosphatases (PTPs) is critical in the regulation of the tyrosine phosphorylation states of specific target proteins. Several PTPs, including leukocyte common antigen related (LAR) phosphatase, PTP-α, PTP-1B and SHPTP2, are highly expressed in insulin-sensitive tissues such as the liver, skeletal muscle, and adipose tissue [1]. These enzymes are all involved in insulin signaling. A number of studies have reported the close involvement of several PTPs, such as LAR, RPTPμ, PTP-RQ, SHP-2, and PTP-BL, in white adipocyte differentiation [19]–[23]. Recently, we performed a PTP profiling analysis of the action of a brown preadipocyte cell line, HIB-1B, during brown adipogenesis. Several PTPs were thought to be involved in brown adipogenesis as well as white adipogenesis [24]. In this study, we found that DUSP10 is involved in brown adipocyte differentiation in mouse brown preadipocytes via the modulation of the p38 phosphorylation level.

## Materials and Methods

### Ethics Statement

All procedures used in animal experiments were performed according to a protocol approved by the Animal care and Use committee of the Korea Research Institute of Bioscience and Biotechnology (KRIBB).

### Reagents and Antibodies

The pharmacological p38 inhibitor, SB203580, was acquired from Calbiochem. Antibodies for p38 (cat. no. 9212), phospho-p38 (p-p38; Thr-180/Tyr-182; cat. no. 9211) and DUSP10 (cat. no. 3483) were purchased from Cell Signaling. Anti-α-tubulin antibody was obtained from Sigma, and secondary antibodies were obtained from Abcam.

### Cell Isolation, Culture, and Brown Adipocyte Differentiation

Brown preadipocytes were obtained from the interscapular BAT of mice (age, late fetal to post-natal days 1–2) and isolated by collagenase dispersion as described previously [25]; [26]. Isolated cells were cultured in a growth medium (high glucose Dulbecco’s Modified Eagle’s Medium [DMEM] containing 1% antibiotic-antimycotic solution and 20% fetal bovine serum [FBS]; Gibco-Invitrogen) at 37°C in a humidified atmosphere with 5% CO_2_. Primary brown preadipocytes were induced to differentiate into mature brown adipocytes, as described previously [27]. Briefly, on day 2 after confluence (day 0), cells were placed in a differentiation medium consisting of DMEM, 10% FBS, and a differentiation cocktail (0.5 mM isobutylmethylxanthine [IBMX], 0.5 µM dexamethasone, 20 nM insulin, 125 µM indomethacin and 1 nM 3,3′,5-triiodo-L-thyronine [T_3_] [all from Sigma]). They were then switched to a maintenance medium composed of DMEM, 10% FBS, 1 nM T_3_, and 20 nM insulin. The medium was replenished every 2 days.

### Overexpression of DUSP10 in Primary Brown Preadipocytes

To ensure that DUSP10 was stably expressed, a retrovirus-mediated infection system was used. The *Dusp10* gene was inserted into the multi-cloning site of the pRetroX-IRES-ZsGreen1 vector (Clontech). The catalytically inactive mutant of *Dusp10*, in which Cys-408 was replaced by Ser, was constructed by site-directed mutagenesis in plasmids [28]. Retroviruses were subsequently produced by transiently co-transfecting GP2-293 cells with a retroviral vector and the VSV-G plasmid using Lipofectamine 2000 (Gibco-Invitrogen). At 48 h after transfection, media containing retroviruses were collected, filtered with 0.45-µm filters, and used to infect cells in the presence of polybrene (8 µg/mL). Infected cells were selected using fluorescence-activated cell sorting (FACS; FACSAria cell sorter, BD Biosciences) and were further maintained in a growth medium, as described previously [26]; [29].

### Gene Silencing Using shRNA

The pSIREN-RetroQ-DsRed Express retroviral vector (Clontech) was employed to knockdown DUSP10 in primary brown preadipocytes. Short-hairpin RNAs (shRNAs) were designed by selecting a target sequence specific to the mouse *Dusp10* gene, as described by Sigma-Aldrich, and thus a sequence for inhibition of DUSP10 expression was obtained (F: 5′-GATCCGTGGTATTGCAGTTAGGTTAAATTCAAGAGATTTAACCTAACTGCAATACCATTTTTTG-3′, R: 5′-AATTCAAAAAATGGTATTGCAGTTAGGTTAAATCTCTTGAATTTAACCTAA.

CTGCAATACCACG-3′). Following this, shRNA sequences were annealed and subcloned according to the manufacturer’s recommendations. The knockdown efficiency was approximately 50% in mouse primary brown preadipocytes. Non-targeting control shRNA (scrambled; SCR) was obtained from Clontech.

### Quantitative Real-time RT-PCR and RT-PCR

Total RNA was extracted from cultured cells with QIAzol lysis reagent (Qiagen, Hilden, Germany), according to the manufacturer’s instructions. First-strand complementary DNA (cDNA) was synthesized using 2 µg of total RNA as the template, 500 ng of random primers, and cDNA synthesis kit components (Promega) in a total volume of 25 µL in accordance with the manufacturer’s recommendations [30]; [31]. The targeted fragment of cDNA for each of the brown adipocyte differentiation-associated genes (Table 1) was amplified by PCR with 2 µL of the reverse transcription (RT) product, 10 pmol of each primer, and a PCR premix (Intron, Daejeon, Korea).

**Table 1:** Primer sequences and PCR conditions used for assessing the mRNA levels of various target proteins by means of qPCR.

Genes	Sequences (5′→3′)	Amplicon (bp)	Annealing Temp. (°C)	Melting Temp. (°C)
UCP1	F : 5′-CTTTGCCTCACTCAGGATTGG-3′	123	60	81.5
	R : 5′-ACTGCCACACCTCCAGTCATT-3′			
PGC-1α	F : 5′-CCCTGCCATTGTTAAGACC-3′	161	60	81.0
	R : 5′-TGCTGCTGTTCCTGTTTTC-3′			
PRDM16	F : 5′-CTTCATGAGCATGCAGGAGA-3′	150	64	84.0
	R : 5′-GACTTTGGCTCAGCCTTGAC-3′			
DUSP10	F : 5′-TTCCTGTTCCTCGGCAATGA-3′	141	60	83.2
	R : 5′-TGGCAGCCTCTTGTAGTTGA-3′			
PPARγ	F : 5′-CAAGAATACCAAAGTGCGATCAA-3	69	60	75.5
	R : 5′-GAGCTGGGTCTTTTCAGAATAATA-3′			
β-actin	F : 5′-GATCTGGCACCACACCTTCT-3′	138	60	83.6
	R : 5′-GGGGTGTTGAAGGTCTCAAA-3′			
TBP	F : 5′-CCCCTTGTACCCTTCACCAAT-3′R : 5′-GAAGCTGCGGTACAATTCCAG-3′	89	60	79.0

### Oil Red-O Staining

Lipid droplets of differentiating or mature brown adipocytes were stained using the Oil red-O (Sigma) staining method, as described previously [19]–[21], [25]. For a quantification analysis, Oil red-O staining dye was extracted and quantified, as described previously [26]; [29].

### Statistical Analysis

Experimental differences were examined for statistical significance using Student’s *t*-test. *P* values that exceeded 0.05 were regarded as statistically significant.

### Quantification of Mitochondrial DNA

Mitochondrial DNA (mtDNA) in mature brown adipocytes was quantified using quantitative real-time PCR (qPCR) and Mito-Tracker fluorescence images. NADH dehydrogenase (ND1) primers were used to evaluate the mitochondrial DNA (F: 5′-GGATCCGAGCATCTTATCCA-3′, R: 5′-GGTGGTACTCCCGCTGTAAA-3′). The ND1 expression level was normalized to TBP (TATA box binding protein) (F: 5′-CCCCTTGTACCCTTCACCAAT -3′, R: 5′-GAAGCTGCGGTACAATTC CAG-3′) [32]; [33]. To label the mitochondria, cells were incubated with Mito-Tracker probes (Invitrogen). The cells were fixed and permeabilized, after which 100 nM of a prewarmed (37°C) staining solution containing Mito-Tracker probes was applied. The cells were incubated in this solution for 30 min. After staining was complete, the solution was replaced with fresh prewarmed media. The cells were fixed with 3.7% formaldehyde in a complete growth medium at 37°C for 15 min. After fixation, they were rinsed 5 times in PBS.

## Results

### DUSP10 is Continuously Upregulated during Brown Adipocyte Differentiation

**Figure 1:**
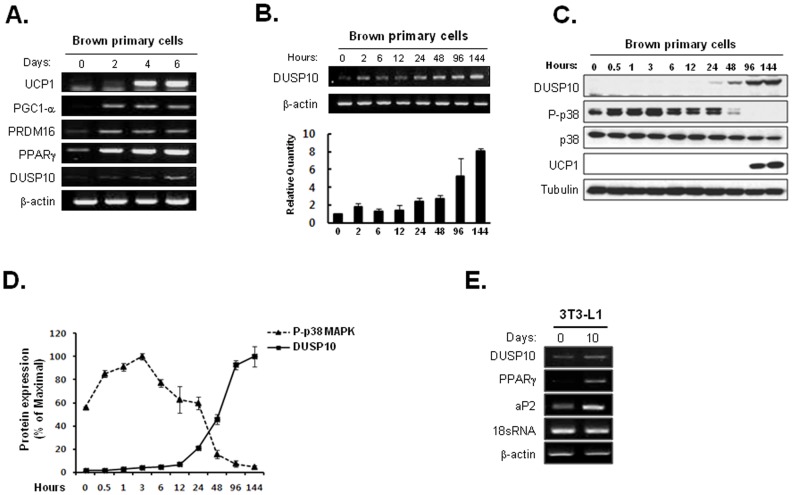
Increased DUSP10 expression levels during the brown adipogenesis of primary cultured cells. **A.** At days 0, 2, 4, and 6 after brown adipogenic induction, the level of DUSP mRNA was assessed by a RT-PCR analysis, and brown adipocyte differentiation was confirmed by measuring the levels of several brown adipogenic marker proteins. **B.** The level of DUSP mRNA was checked using RT-PCR at various time points, especially during the early stage of differentiation. **C.** DUSP10 and p38 protein levels were analyzed with western blot analysis. **D.** Western blot data of the DUSP10 and active p38 levels were quantified using Image J software. **E.** mRNA profiling analysis of DUSP10 during the white adipogenesis of 3T3-L1 preadipocytes.

To determine the functional roles of the PTP family during brown adipocyte differentiation, a PTP expression profiling analysis was performed for primary brown preadipocytes. The primary cells were harvested during brown adipogenesis (days 0, 2, 4, and 6 after culturing with a brown adipogenic medium), and the mRNA levels were measured. As shown in Fig. 1A, the levels of brown adipocyte differentiation marker proteins such as PPARγ, UCP1, PRDM16 and PGC1-α, were significantly upregulated during brown adipogenesis. This result clearly indicates that a substantial amount of brown adipocyte differentiation occurred under our experimental conditions. Among several PTPs showing significant alterations of thier mRNA levels, the DUSP10 mRNA levels dramatically increased after the addition of the differentiation cocktail (Fig. 1A). In particular, the expression level of the DUSP10 protein increased continuously, with marked upregulation at a late stage of differentiation (Fig. 1B). Additionally, western blot analysis data clearly showed that DUSP10 was significantly upregulated 24 h after differentiation (Figs. 1C and 1D). Furthermore, the brown adipogenesis-specific differential expression pattern was detected in the case of DUSP10 [34]. Thus, we considered that the fine regulation of DUSP10 may allow some control during brown adipogenesis.

### Phosphorylation of p38 MAPK Drastically Increased Early during Brown Adipocyte Differentiation

DUSP10 (also known as MKP-5) inactivates its target kinases by dephosphorylating both the phosphoserine/threonine and phosphotyrosine residues. DUSP10 binds to and inactivates p38 and the N-terminal kinase, JNK, but not ERK [35]. Therefore, we checked the active p38 and JNK levels during brown adipocyte differentiation. As shown in Fig. 1C, the p-p38 (active p38) levels were significantly decreased at 48 h after adipogenic differentiation. In contrast, at the same time, DUSP10 was dramatically upregulated (Fig. 1D). In the case of JNK, we did not detect significant changes during brown adipocyte differentiation (data not shown).

Next, to test if the upregulation of DUSP10 was a phenomenon specific to brown adipogenesis, we conducted an expression analysis of DUSP10 during the white adipogenesis of 3T3-L1 preadipocytes. As shown in Fig. 1E, there was no dramatic alteration in the expression of DUSP10 during white adipogenesis.

### Overexpression of DUSP10 Inhibits Brown Adipocyte Differentiation

**Figure 2:**
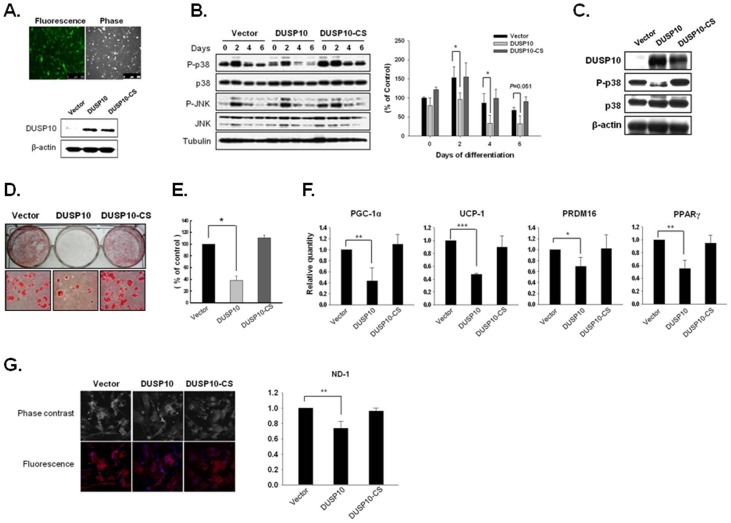
The Overexpression of DUSP10 inhibits brown adipocyte differentiation. **A.** GFP expression was directly monitored using fluorescence microscopy after enrichment with FACS. In addition, the overexpression of DUSP10 and the catalytic inactive DUSP10 mutant was confirmed by western blot analysis. **B.** The expression levels of p38, phospho-p38 (p-p38), JNK and phosphor-JNK (p-JNK) during differentiation after DUSP10 or DUSP10-CS ectopic expression were assessed by western blot analysis (left panel). Western blot data were quantified using Image J software (right panel; *P<0.05). **C.** The overexpression of DUSP10 and the mutant DUSP10 at a late stage (day 6) was confirmed by western blot analysis. **D.** Primary cultured cells were stained with Oil red O to visualize lipid droplets upon 6 days of differentiation. **E.** Quantification of stained cells was performed using a dye-extraction buffer (*P<0.05). **F.** The expression levels of brown specific adipogenic markers were analyzed using qPCR after DUSP10 overexpression (*P<0.05; **P<0.01; ***P<0.001). **G.** MitoTracker fluorescence (Invitrogen) was measured using confocal microscopy. MitoTracker is identified by the red color, and DAPI-stained nuclei are blue. The mitochondrial DNA (mtDNA) content was quantified by qPCR (**P<0.01).

Next, we infected primary brown preadipocytes with *Dusp10* by using a retroviral system (*Dusp10*-IRES-GFP) to clarify the functional role of DUSP10 in brown adipogenesis. *Dusp10*-CS (catalytic inactive mutant) and a control vector were used as negative controls. Infected cells were enriched using a FACS sorter. Most of the cells were found to be GFP positive upon examination by fluorescence microscopy, and the overexpression of wild-type and mutant DUSP10 was confirmed by a western blot analysis (Fig. 2A). DUSP10 overexpression induced a reduction in active p38 levels, especially in the early stages of differentiation (Fig. 2B), and this expression was continuously detected during the latter stages (6 days) (Fig. 2C). P-p38 was downregulated upon DUSP10 expression until the late stages. On the other hand, there were no significant expression changes of p-JNK and JNK upon DUSP10 ectopic expression. The infected cells were induced to differentiate into mature brown adipocytes, and then Oil red-O staining was performed to assess the lipid accumulation 6 days after culturing with a differentiation medium (Fig. 2D). DUSP10 overexpression led to a lower degree of lipid accumulation than that noted with the control and DUSP10 mutant (Figs. 2D and 2E). The expression levels of brown adipogenic markers, such as PGC-1α,RRDM16 and UCP1, were consistently and significantly reduced upon DUSP10 overexpression (Fig. 2F). One typical difference between white and brown adipocytes is the mitochondrial activity and number of mitochondria, both of which are higher in brown adipocytes. Thus, the differentiated adipocyte cells were stained with MitoTracker. The result clearly revealed a dramatic decrease in staining upon DUSP10 overexpression (Fig. 2G). On the other hand, the DUSP10-CS mutant (catalytic inactive mutant) demonstrated no significant changes during the phosphorylation of p38, lipid-drop accumulation, the expression of brown adipogenic markers, and the mitochondrial content, indicating that phosphatase activity is critical in decreasing brown adipocyte differentiation.

### Use of the p38 Inhibitor at an Early Stage Induced a Reduction in Differentiation, which Recovered after DUSP10 Knockdown

**Figure 3:**
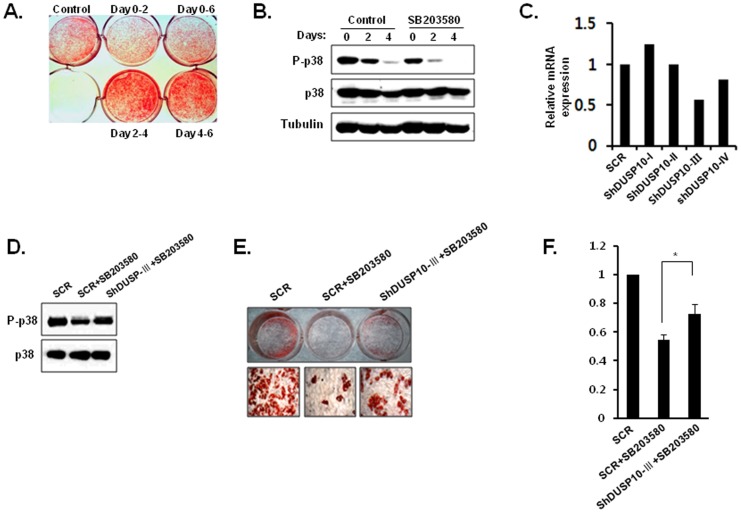
P38 inhibitor treatment at an early stage of brown adipogenesis induced the suppression of differentiation. **A.** SB203580 (p38 inhibitor) was added to the culture medium at different time points during brown adipogenesis. After 6 days of differentiation, lipid accumulation was measured in fixed cells by Oil red O staining, and the quantification of stained cell was performed using dye-extraction buffer (*P<0.05). **B.** P-p38 levels during treatment with SB203580 were analyzed by western blot analysis. **C.** The knockdown effect of shRNA constructs against DUSP10 was confirmed by qPCR. **D.** P-p38 levels recovered after the introduction of the DUSP10-III shRNA construct in SB203580-treated cells and were analyzed using western blot analysis. **E.** Accumulated lipid droplets were stained with Oil red O after 6 days of differentiation. **F.** Stained lipid droplets were quantified using a dye-extraction method (*P<0.05).

As shown in Fig. 3A, the application of a p38 inhibitor, SB203580, at an early stage (0–2 days) was sufficient to cause the suppression of differentiation. On the other hand, exposure durng the terminal stage (4–6 days) did not cause any significant changes in brown adipocyte differentiation compared to that in control cells (Fig. 3A). Additionally, the application of SB203580 induced a reduction in the levels of p-p38 (Fig. 3B). Next, we tested whether the suppression of brown adipocyte differentiation by a SB203580 treatment is recovered by the knockdown of DUSP10. To do this, several shRNA constructs were produced and tested for their effectiveness in reducing DUSP10 expression in primary brown preadipocytes using a retrovirus expression system (Clontech, pSIREN-RetroQ-DsRed). The infected cells were enriched by FACS. Following this, the knockdown of endogenous DUSP10 expression was additionally confirmed by qPCR, which showed that the shRNA DUSP10-III construct reduced endogenous DUSP10 expression (Fig. 3C). The application of SB203580 to primary brown preadipocyte cells infected with shRNA DUSP10-III showed partially recovered p-p38 levels (Fig. 3D). Retrovirally transduced brown preadipocytes were induced to differentiate into brown adipocytes. As shown in Figs. 3E and 3F, the knockdown of DUSP10 significantly induced the partial recovery of reduced brown adipocyte differentiation due to the treatment with SB203580. These results suggest that the activation of p38 is critical at an early stage of brown adipogenic differentiation and that DUSP10 is involved in the inactivation of p38 at an appropriate time point of brown adipocyte differentiation.

## Discussion

White and brown adipocytes were previously believed to derive from the same precursor cells. However, recent studies have shown that brown adipocytes have an origin that is distinct from that of white adipocytes. Thus, it is conceivable that there are significant mechanistic differences between white and brown adipogenic differentiation [1]. Most studies of adipogenesis have focused on the differentiation of white adipocytes, but the molecular differentiation mechanism of brown adipocytes has still not been extensively investigated [1]; [26]; [36]; [37].

In this study, we performed a PTP profiling analysis during the brown adipogenesis of mouse primary brown preadipocytes. As expected, the results obtained from the PTP profiling analysis of brown adipocyte differentiation were significantly different from those for white adipocyte differentiation (data not shown). Among several PTPs identified as being differentially expressed during brown adipogenesis, we focused on the MAPK phosphatase (MKP) DUSP10. DUSP10 (also known as MKP-5) acts as a negative regulator of the stress-activated p38 and JNK MAPK kinases [35]. DUSP10 is widely expressed in various tissues, and its subcellular localization is unique. DUSP10 is evenly distributed both in the cytoplasm and in the nucleus (from GeneCard^®^, http//www.genecards.org). MAPKs are key intracellular signaling pathways that play critical roles in many essential processes, including adipocyte differentiation. Thus, MKPs are subjected to tight regulation [38]; [39]. The expression levels of DUSP10 were found to increase continuously during brown adipocyte differentiation. In particular, dramatic upregulation of the DUSP10 protein was detected 24 h after differentiation. On the other hand, the level of the active p38 (p-p38) protein is reciprocally reduced with that of DUSP10. The ectopic expression of DUSP10 in primary brown preadipocyte cells significantly suppressed brown adipogenesis, implying that DUSP10 is crucial for brown adipocyte differentiation. Additionally, DUSP10 overexpression, but not the introduction of the DUSP10-CS catalytic inactive mutant, induced a reduction in adipogenic marker proteins, such as PGC-1α, UCP1, PRDM16 and PPARγ. The active p38 level is also reduced upon DUSP10 overexpression. Other PTPs known as negative regulators of p38, such as DUSP-1, DUSP-2, DUSP-4, DUSP-8, DUSP-9, and DUSP-16, showed no significant expression changes during brown adipocyte differentiation [34]. DUSP10 overexpression also had no effect on JNK activation (Fig. 2B). Therefore, these results strongly suggest that DUSP10 is a potent anti-adipogenic regulator that works by modulating the phosphorylation levels of p38 during brown adipocyte differentiation. Next, we used a specific inhibitor of active p38, SB203580, to confirm the importance of DUSP10 by controlling p38 in brown adipogenesis. As shown in Fig. 3A, the presence of SB203580 in the first 0–2 days led to the suppression of brown adipocyte differentiation, with little change occurring after these 2 days up to day 6. This finding strongly suggests that active p38 is a critical factor at an early stage of brown adipocyte differentiation. The suppression effect of SB203580 was found to be partially recovered by the knockdown of DUSP10. In conjunction with earlier reports, the results presented here provide conclusive evidence that active p38 is essential in the early stages of brown adipogenesis and that DUSP10 dephosphorylates and inactivates p38. Thus, DUSP10 downregulation by a specific inhibitor can increase brown adipogenesis and subsequently causes an anti-obesity effect via increased heat generation at the expense of ATP. DUSP10 could be a valuable novel target for the treatment of obesity.

## References

[1] Bae K-H, Kim WK, Lee SC (2012) Involvement of protein tyrosine phosphatases in adipogenesis: New anti-obesity targets?. BMB Rep 45: 700–706. 23261055 10.5483/BMBRep.2012.45.12.235 PMC4133817

[2] Rosen ED, Spiegelman BM (2006) Adipocytes as regulators of energy balance and glucose homeostasis. Nature 444: 847–853. 17167472 10.1038/nature05483 PMC3212857

[3] Fruhbeck G, Becerril S, Sáinz N, Garrastachu P, García-Velloso MJ (2009) BAT: a new target for human obesity?. Trends Pharmacol Sci 8: 387–396. 10.1016/j.tips.2009.05.003 19595466

[4] Cypess AM, Lehman S, Williams G, Tal I, Rodman D, et al (2009) Identification and Importance of Brown Adipose Tissue in Adult Humans. N Eng J Med 360: 1509–1517. 10.1056/NEJMoa0810780 PMC2859951 19357406

[5] Zingaretti MC, Crosta F, Vitali A, Guerrieri M, Frontini A, et al (2009) The presence of UCP1 demonstrates that metabolically active adipose tissue in the neck of adult humans truly represents brown adipose tissue. FASEB J 23: 3113–3120. 19417078 10.1096/fj.09-133546

[6] Virtanen KA, Lidell ME, Orava J, Heglind M, Westergren R, et al (2009) Functional brown adipose tissue in healthy adults. N Eng J Med 360: 1518–1525. 10.1056/NEJMoa0808949 19357407

[7] Zhang W, Liu HT (2002) MAPK signal pathways in the regulation of cell proliferation in mammalian cells. Cell Res 12: 9–18. 11942415 10.1038/sj.cr.7290105

[8] Ichijo H (1999) From receptors to stress-activated MAP kinases. Oncogene 18: 6087–6097. 10557099 10.1038/sj.onc.1203129

[9] Obata T, Brown GE, Yaffe MB (2000) MAP kinase pathways activated by stress: the p38 MAPK pathway. Crit Care Med 28: 67–77. 10807318 10.1097/00003246-200004001-00008

[10] Raingeaud J, Gupta S, Rogers JS, Dickens M, Han J, et al (1995) Pro-inflammatory cytokines and environmental stress cause p38 mitogen-activated protein kinase activation by dual phosphorylation on tyrosine and threonine. J Biol Chem 270: 7420–7426. 7535770 10.1074/jbc.270.13.7420

[11] Engelman JA, Berg AH, Lewis RY, Lin A, Lisanti MP, et al (1999) Constitutively active mitogen-activated protein kinase kinase 6 (MKK6) or salicylate induces spontaneous 3T3-L1 adipogenesis. J Biol Chem 274: 35630–35638. 10585441 10.1074/jbc.274.50.35630

[12] Engelman JA, Lisanti MP, Scherer PE (1998) Specific inhibitors of p38 mitogen-activated protein kinase block 3T3-L1 adipogenesis. J Biol Chem 273: 32111–32120. 9822687 10.1074/jbc.273.48.32111

[13] Hata K, Nishimura R, Ikeda F, Yamashita K, Matsubara T, et al (2003) Differential roles of Smad1 and p38 kinase in regulation of peroxisome proliferator-activating receptor gamma during bone morphogenetic protein 2-induced adipogenesis. Mol Biol Cell 14: 545–555. 12589053 10.1091/mbc.E02-06-0356 PMC149991

[14] Tseng Y-H, Kokkotou E, Schulz TJ, Huang TL, Winnay JN, et al (2008) New role of bone morphogenetic protein 7 in brown adipogenesis and energy expenditure. Nature 454: 1000–1004. 18719589 10.1038/nature07221 PMC2745972

[15] Bordicchia M, Liu D, Amri EZ, Ailhaud G, Dessì-Fulgheri P, et al (2012) Cardiac natriuretic peptides act via p38 MAPK to induce the brown fat thermogenic program in mouse and human adipocytes. J Clin Invest 122: 1022–1036. 22307324 10.1172/JCI59701 PMC3287224

[16] Mustelin T, Abraham RT, Rudd CE, Alonso A, Merlo JJ (2002) Protein tyrosine phosphorylation in T cell signaling. Front Biosci 7: 918–969. 10.2741/A821 11897562

[17] Alonso A, Sasin J, Bottini N, Friedberg I, Friedberg I, et al (2004) Protein tyrosine phosphatases in the human genome. Cell 117: 699–711. 15186772 10.1016/j.cell.2004.05.018

[18] Mustelin T, Taskén K (2003) Positive and negative regulation of T-cell activation through kinases and phosphatases. Biochem J 371: 15–27. 12485116 10.1042/BJ20021637 PMC1223257

[19] Kim WK, Jung H, Kim DH, Kim EY, Chung JW, et al (2009) Regulation of adipogenic differentiation by LAR tyrosine phosphatase in human mesenchymal stem cells and 3T3-L1 preadipocytes. J Cell Sci 122: 4160–4167. 19910497 10.1242/jcs.053009

[20] Kim WK, Jung H, Kim EY, Kim DH, Cho YS, et al (2011) RPTPμ tyrosine phosphatase promotes adipogenic differentiation via modulation of p120 catenin phosphorylation. Mol Biol Cell 22: 4883–4891. 21998202 10.1091/mbc.E11-03-0175 PMC3237630

[21] Jung H, Kim WK, Kim DH, Cho YS, Kim SJ, et al (2009) Involvement of PTP-RQ in differentiation during adipogenesis of human mesenchymal stem cells. Biochem Biophys Res Comm 383: 252–257. 19351528 10.1016/j.bbrc.2009.04.001

[22] Uehara T, Suzuki K, Yamanaka H, Kizaki T, Sakurai T, et al (2007) SHP-2 positively regulates adipogenic differentiation in 3T3-L1 cells. Int J Mol Med 19: 895–900. 17487421

[23] Glondu-Lassis M, Dromard M, Chavey C, Puech C, Fajas L, et al (2009) Downregulation of protein tyrosine phosphatase PTP-BL repress adipogenesis. Int J Biochem Cell Biol 41: 2173–2180. 19782949 10.1016/j.biocel.2009.04.004

[24] Choi H-R, Kim WK, Kim EY, Jung H, Kim J-H, et al (2012) Protein tyrosine phosphatase profiling analysis of HIB-1B cells during brown adipogenesis. J Microbiol Biotechnol 22: 1029–1033. 22580324 10.4014/jmb.1112.12059

[25] Klein J, Fasshauer M, Ito M, Lowell BB, Benito M, et al (1999) β(3)-adrenergic stimulation differentially inhibits insulin signaling and decreases insulin-induced glucose uptake in brown adipocytes. J Biol Chem 274: 34795–34802. 10574950 10.1074/jbc.274.49.34795

[26] Kim WK, Choi H-R, Park SG, Ko Y, Bae K-H, et al (2012) Myostatin inhibits brown adipocyte differentiation via regulation of Smad3-mediated β-catenin stabilization. Int J Biochem Cell Biol 44: 327–334. 22094186 10.1016/j.biocel.2011.11.004

[27] Tseng YH, Kriauciunas KM, Kokkotou E, Kahn CR (2004) Differential roles of insulin receptor substrates in brown adipocyte differentiation. Mol Cell Biol 24: 1918–1929. 14966273 10.1128/MCB.24.5.1918-1929.2004 PMC350563

[28] Tonoue T, Moriguchi T, Nishida E (1999) Molecular cloning and characterization of a novel dual specificity phosphatase, MKP-5. J Biol Chem 274: 19949–199456. 10391943 10.1074/jbc.274.28.19949

[29] Kim EY, Kim WK, Kang HJ, Kim J-H, Chung SJ, et al (2012) Acetylation of malate dehydrogenase 1 promotes adipogenic differentiation via activating its enzymatic activity. J Lipid Res 53: 1864–1876. 22693256 10.1194/jlr.M026567 PMC3413227

[30] Jang M, Park BC, Kang S, Chi S-W, Cho S, et al (2009) Far upstream element-binding protein-1, a novel caspase substrate, acts as a cross-talker between apoptosis and the *c-myc* oncogene. Oncogene 28: 1529–1536. 19219071 10.1038/onc.2009.11

[31] Kim WK, Cho HJ, Ryu SI, Hwang H-R, Kim D-H, et al (2008) Comparative proteomic analysis of peripheral blood mononuclear cells from atopic dermatitis patients and healthy donors. BMB Rep 41: 597–603. 18755076 10.5483/bmbrep.2008.41.8.597

[32] Seal P, Kajimura S, Yang W, Chin S, Rohas LM, et al (2007) Transcriptional control of brown fat determination by PRDM16. Cell Metab 6: 38–54. 17618855 10.1016/j.cmet.2007.06.001 PMC2564846

[33] Mori H, Prestwich TC, Reid MA, Longo KA, Gerin I, et al (2012) Secreted frizzled-related protein 5 suppresses adipocyte mitochondrial metabolism through WNT inhibition. J Clin Invest 122: 2405–2416. 22728933 10.1172/JCI63604 PMC3386832

[34] Choi H-R, Kim WK, Park A, Jung H, Han BS, et al.. (2013) Protein tyrosine phosphatase profiling studies during brown adipogenc differentiation of mouse primary brown preadipocytes. BMB Rep in press. 10.5483/BMBRep.2013.46.11.058 PMC4133841 24152912

[35] Owens DM, Keyse SM (2007) Differential regulation of MAP kinase signaling by dual-specificity protein phosphatases. Oncogene 26: 3203–3213. 17496916 10.1038/sj.onc.1210412

[36] Rosen ED, MacDougald OA (2006) Adipocyte differentiation from the inside out. Nat Rev Mol Cell Biol 7: 885–896. 17139329 10.1038/nrm2066

[37] Vila-Bedmar R, Lorenzo M, Fernández -Veledo S (2010) Adenosine 5′-monophosphate-activated protein kinase-mammalian target of rapamycin cross talk regulates brown adipocyte differentiation. Endocrinology 151: 980–992. 20133456 10.1210/en.2009-0810

[38] Bermudez O, Pages G, Gimond C (2010) The dual-specificity MAP kinase phosphatases: critical roles in development and cancer. Am J Physiol Cell Physiol 299: C189–C202. 20463170 10.1152/ajpcell.00347.2009

[39] Bost F, Aouadi M, Binetruy CB (2005) The role of MAPKs in adipocyte differentiation and obesity. Biochimie 87: 51–56. 15733737 10.1016/j.biochi.2004.10.018

